# Integrated Computational Approach for Virtual Hit Identification against Ebola Viral Proteins VP35 and VP40

**DOI:** 10.3390/ijms17111748

**Published:** 2016-10-26

**Authors:** Muhammad Usman Mirza, Nazia Ikram

**Affiliations:** 1 Centre for Research in Molecular Medicine (CRiMM), The University of Lahore, Defense Road, Lahore 54000, Pakistan; 2 Institute of Molecular Biology and Biotechnology, The University of Lahore, Lahore 54000, Pakistan; naxiaikram@gmail.com

**Keywords:** Ebola virus, phytochemicals, molecular docking, VP35, VP40, retrospective validation, virtual screening

## Abstract

The Ebola virus (EBOV) has been recognised for nearly 40 years, with the most recent EBOV outbreak being in West Africa, where it created a humanitarian crisis. Mortalities reported up to 30 March 2016 totalled 11,307. However, up until now, EBOV drugs have been far from achieving regulatory (FDA) approval. It is therefore essential to identify parent compounds that have the potential to be developed into effective drugs. Studies on Ebola viral proteins have shown that some can elicit an immunological response in mice, and these are now considered essential components of a vaccine designed to protect against Ebola haemorrhagic fever. The current study focuses on chemoinformatic approaches to identify virtual hits against Ebola viral proteins (VP35 and VP40), including protein binding site prediction, drug-likeness, pharmacokinetic and pharmacodynamic properties, metabolic site prediction, and molecular docking. Retrospective validation was performed using a database of non-active compounds, and early enrichment of EBOV actives at different false positive rates was calculated. Homology modelling and subsequent superimposition of binding site residues on other strains of EBOV were carried out to check residual conformations, and hence to confirm the efficacy of potential compounds. As a mechanism for artefactual inhibition of proteins through non-specific compounds, virtual hits were assessed for their aggregator potential compared with previously reported aggregators. These systematic studies have indicated that a few compounds may be effective inhibitors of EBOV replication and therefore might have the potential to be developed as anti-EBOV drugs after subsequent testing and validation in experiments in vivo.

## 1. Introduction

*Filoviridae*, from the EBOV family, is a lipid-enveloped, negative-strand RNA virus that causes Ebola haemorrhagic disease, characterised by fever and an ensuing bleeding diathesis that has high mortality in both humans and non-human primates [1]. Although EBOV has been acknowledged for nearly 40 years, the recent outbreak in West Africa has created an appalling situation in the region. The outbreak started in March 2014 in the Republic of Guinea, and has continued to spread throughout Sierra Leone and Liberia, where it is reported to be one of the largest outbreaks in history [2,3]. Mortality rates range from 53% to as high as 90%, with a total of 7178 cases reported until 1 October 2014 [4,5].

**Figure 1:**
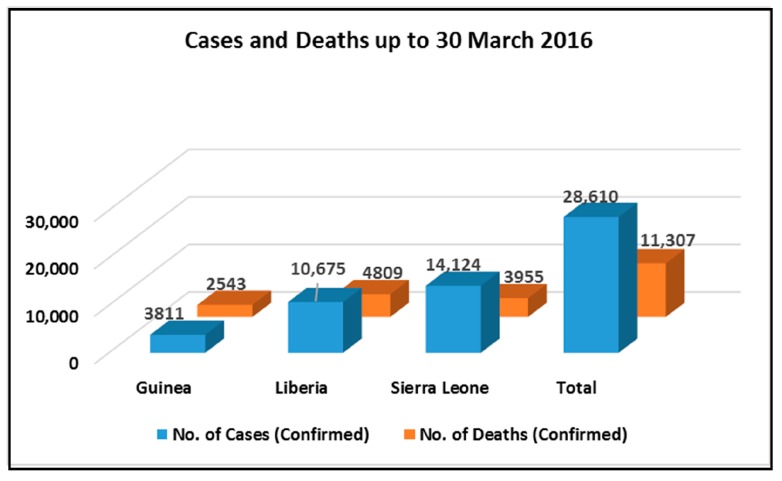
Ebola situation report March 2016. Ebola virus disease cases and deaths. Data are based on official information reported by the Ministry of Health in an Ebola situation report published on 30 March 2016.

As of 30 March 2016, 28,610 suspected cases and 11,307 deaths have been reported in the most affected countries of Guinea, Liberia, and Sierra Leone (Ebola Situation Report, March 2016) (Figure 1). Although the virus is already on the verge of becoming a socioeconomic burden, no vaccine or treatment has succeeded in controlling or treating the disease. The *Ebola virus* genus comprises five species, each named after the location where it was first identified: *Zaire* (EBOV-Z), *Sudan* (EBOV-S), *Taï Forest* (EBOV-T), *Bundibugyo* EBOV-B, and *Reston* (EBOV-R), with varying fatality rates [6]. A vaccine against EBOV-Z has shown the potential of immune responses against surface glycoproteins and nucleoproteins [7]. Several investigations into anti-Ebola drugs have been carried out, but no effective drug has yet been approved by the FDA. Profectus Bioscience, Inc., a clinical-stage vaccine development company, has recently developed VesiculoVax, a vectored Ebola virus vaccine, which is currently in phase I clinical trials [8]. Several experimental drugs have also been tested against Ebola, including a synthetic adenosine analogue (BCX4430) developed by BioCryst, which is reported to inhibit filoviruses in humans [9]. This drug inhibits viral RNA polymerase activity by functioning as a non-obligate RNA chain terminator. Tests against EBOV and the Marburg virus have been performed in rodents, achieving promising results. However, tests have not yet been carried out in humans. There have also been positive developments with ZMapp, which is a combination of two individual monoclonal antibodies, MB-003 (Mapp) and ZMAb (Defyrus/PHAC), from a strain of the tobacco plant. This treatment has been assessed in animals with a 43% success rate; however, as with others, it has not yet been tested in humans [10]. One treatment regime, CMX001, was approved by the FDA and administered to two Ebola patients in 2014. One patient, being critically ill, died, while the other survived and was declared Ebola-free. However, in early 2015, trials ended as the manufacturer withdrew support. Another treatment regime, T-705 (favipiravir), when tested on mammalian cells, proved to be non-toxic [11]. In 2014, results of a clinical trial with this drug suggested a decrease in the mortality of Ebola patients affected with lower levels of Ebola virus, while patients with higher Ebola virus levels remained unaffected. Treatment with FGI-106, similar to T-705, had curative effects on the Ebola virus when tested on animals. Ribavirin, a prodrug known for treating a variety of viruses, was reported to have poor results against the Ebola virus [12]. Another drug, Lamivudine, was tested on 15 Ebola patients and positive results were indicated. Thirteen patients survived and maintained a virus-free state until at least the end of the course. Since being found effective against Ebola in monkeys, TKM-Ebola treatment went into phase I clinical trials at the start of 2014. However, these trials were partially suspended by the FDA due to adverse reactions with this drug, and in March 2015, TKM-Ebola phase II clinical trials were stopped since statistical analysis indicated a lack of progress. Triazavirin is currently being tested for its potential against the Ebola virus.

An earlier search for a target-specific drug focused on the viral glycoprotein. It was observed that the glycoprotein is the leading cause of vascular cell injuries, and also leads to direct structural damage to endothelial cells, which in turn triggers a haemorrhagic diathesis [13]. The search has now broadened to other proteins. Investigations on Ebola viral proteins VP24, VP30 and VP35 have shown that an immunological response can be elicited in mice, and these proteins are now considered as critical components of a vaccine designed to protect against Ebola haemorrhagic fever [7,14]. Sequence analysis of the Ebola virus has shown that the organization of the viral genome is similar to that of Rhabdoviruses and Paramyxoviruses. The order of EBOV genes was also predicted as follows: *3′-NP-VP35-VP40-GP-VP30-VP24-L* [15]. VP40, a 326 amino acid viral matrix protein, is the most abundant protein in viral particles and is crucial to virus assembly and budding. The budding process takes place at the plasma membrane after the assembly of VP40 and requires lipid raft microdomains. VP40 is located under the viral bilayer and maintains the structural integrity of the particles [16,17]. It has also been observed that VP40 is essential for viral or host cell RNA metabolism during viral replication [18,19]. We have identified VP40 assemblies in a filamentous structure and have shown that disruption of these structures halts viral egress. Studies on the membrane binding and structural properties of VP40 have revealed potential drug sites [18,20]. The crystal structure of VP40 shows that it is an octamer and forms a pore-like structure that binds RNA. It is a monomeric structure, and both N- and C-terminal domains are associated with the membrane. The RNA protein structure is stabilised by 140 amino acid residues of VP40 (including residues Thr123, Phe125 and Arg134 of a fragment of the N-terminal domain) and UGA (stop codon) of RNA, which is also a potential target for antiviral drug design [14,18].

In the quest for a target site, considerable interest has also been shown in the multi-functional VP35 protein. VP35 is crucial to viral replication and virulence. Its functions include inhibition of IFN-α/β production and protein kinase R, suppression of RNA interference, and, most importantly, being a cofactor for the viral polymerase domain (VP35 IID), responsible for most activities performed by this protein [21,22,23]. The ability of VP35 to suppress RNA silencing enhances virus replication, which makes VP35 another important target in drug design.

There are a few published studies that have identified other compounds against EBOV, as reviewed by De Clercq (2015). Among them, some have highlighted the potential for the repurposing of FDA-approved drugs on different Ebola strains [24,25,26,27,28,29,30,31,32,33]. Although these studies have shown promising results, they were not exhaustive screens of all FDA drugs. It was also unclear whether these drugs had biological activities when used in combination with therapies to overcome drug resistance, as the virus is a highly variable species. Studies in silico have been published for Ebola VP35 and VP40, and, very recently, a machine learning method that uses Bayesian and Support Vector Machine (SVM) algorithms was proposed by Ekin et al. [25] for the identification of novel Ebola inhibitors from already reported antiviral data. Over the past decade, there has been significant interest in the exploitation of phytochemicals for pharmaceutical use, many of which have antiviral or anticancer activities; a few studies have focused on the potential of phytochemicals as possible parental compounds for the treatment of haemorrhagic fever [34,35]. Kolokoltsov et al. demonstrated that a cocktail of genistein and tyrphostin AG1478, both of which are kinase inhibitors, forms a broad spectrum antiviral agent that can be used for the treatment of both *arenavirus* and *filovirus* haemorrhagic fevers [36]. They established that both genistein and tyrphostin individually inhibit the entry of these viruses into cells by interfering with endocytosis and the activity of uncoating proteins. Such positive results confirm the view that the exploration of phytochemicals for EBOV has potential.

A plethora of plant species have been found to produce novel antiviral agents, and a variety of active phytochemicals have been isolated. Testing these compounds against specific viruses to discover potential anti-viral compounds requires exhaustive effort and resources. However, virtual hits can be determined using computational approaches such as molecular docking, virtual screening, and machine learning. Moreover, the toxicity of these compounds can be predicted comparatively quickly with state-of-the-art computational Quantity-Structure-Activity-Relationship (QSAR) analysis.

The current study aimed to identify potential virtual hits from a wide range of credible phytochemical databases using in silico approaches. To our knowledge, no one has attempted to screen nearly 150,000 natural product compounds. This study used different chemoinformatic approaches against Ebola VP35 and VP40, including protein binding site prediction, drug-likeness, ADMET (Absorption, Distribution, Metabolism, Excretion and Toxicity) properties, drug metabolism, drug safety profiling, and molecular docking of a wide range of phytochemicals. In early drug discovery processes through virtual screening, colloidal aggregation of small compounds is a primary source of non-specific inhibition in an assay that can give false positive results [37]. Therefore, the best virtual hits were further analysed to check for their aggregator potential compared with previously reported aggregators. Retrospective validation through enrichment via a Receiver Operating Characteristics (ROC) curve further helped us evaluate virtual hits and docking protocols. Comparative homology modelling of other EBOV strains and structural alignments were performed to map binding site residues of VP35 and VP40 in all Ebola strains under study. This systematic study has produced promising findings that may help combat this deadly disease.

## 2. Results

### 2.1. Binding Site Analysis

A detailed investigation of binding pockets from X-ray determined crystal structures was performed and evaluated through the Computed Atlas of Surface Topography of proteins (CASTp) server. The recent dimeric structure of the VP40 protein reveals that blocking the N- or C-terminal assembly inhibits EBOV assembly. Potential target residues include Thr123, His124, Phe125, Gly126, Arg134, Asn136, Tyr171, and Phe172, of which Thr123, Phe125, and Arg134 are especially crucial for docking studies as these residues interact with RNA [16,18].

NMR mapping and high-resolution X-ray crystal structure of VP35 revealed that small compounds bind to the Ebola Interferon inhibitory domain (eIID), and also to VP35 domains that are essential for the formation of the replication complex through molecular interactions with viral nucleoprotein. There were two basic patches in IID: the first basic patch (FBP) and central basic patch (CBP). The former is important for molecular interactions with the Ebola virus nucleoprotein and VP35 polymerase cofactor function, whilst the latter has its role in VP35 dsRNA binding and inhibition of IFN [38]. It has been shown that Ala221, Arg225, Gln241, Leu242, Lys248, Lys251, Pro293, Ile295, Ile297, Asp302 and Phe328 are located near and inside the FBP groove. Approximately 20 residues from eIID make up the binding pocket and are distributed evenly between the alpha helical and beta sheet subdomains.

### 2.2. In Silico Screening

**Figure 2:**
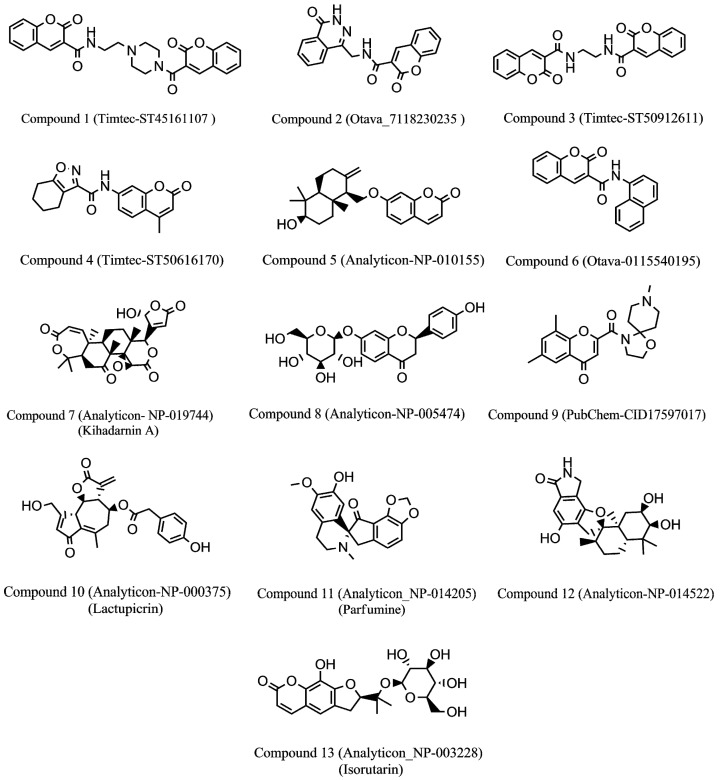
Chemical structures of virtual hits are represented in 2D format.

**Figure 3:**
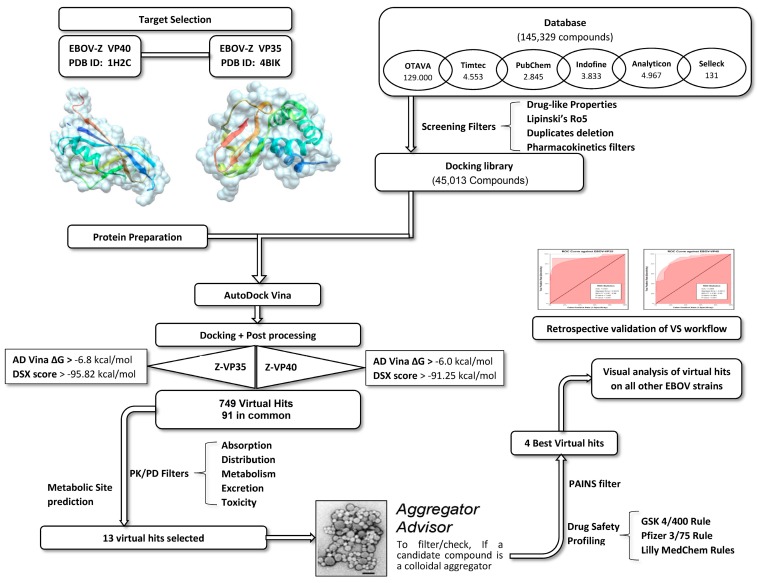
Schematic workflow summarising the screening of Ebola virus (EBOV) inhibitors through a series of steps.

To identify new natural anti-Ebola virtual hits, SBVS of 145,329 compounds and the application of a series of filters led us to select 13 best hits that indicated high binding energies with promising ADMET properties, as shown in Figure 2. These 13 virtual hits were further checked for colloidal aggregator potential, using Aggregator Advisor, and seven virtual hits were finally selected. The schematic representation of chronological virtual screening is shown in Figure 3. The natural product database, containing 145,329 compounds, was built by combining libraries of Drug-like green compounds (OTAVA chemicals), Phytochemical compounds (Pubchem), Natural products (Analyticon Discovery and Selleck) and Flavonoids (Timtec and Indofine Chemical companies). Subsequently, filtering for drug-likeness, Lipinski’s Ro5, Duplicates deletion, and one relevant pharmacokinetics parameters (HIA) led us to consider 45,013 compounds globally. EBOV-Z VP35 and VP40 were used to perform the SBVS procedure because of X-ray resolved structures present in PDB. A total of 45,013 compounds were docked to the FBP groove of IID of EBOV-Z VP35 and the RNA-interacting region of EBOV-Z VP40. The best Autodock Vina docking score for both proteins was considered and subjected to further analysis. In particular, the Molecular Interaction Field (MIF) strategy-based rescoring function, DrugScore eXtended, was employed. In order to set an energy cut-off so as to select the best virtual hits, the same Autodock Vina protocol was carried out for the 10 active compounds that were determined experimentally to inhibit EBOV-Z VP35 by Brown et al. i.e., GA017 (PDB ID: 4IBB), GA246 (PBD ID: 4IBC), VPL27 (PDB ID: 4IBD), VPL29 (PDB ID: 4IBE), VPL42 (PDB ID: 4IBF), VPL48 (PDB ID: 4IBG), VPL51 (PDB ID: 4IBH, Unreleased PDB structure, therefore not included in docking procedure), VPL57 (PDB ID: 4IBI), VPL58 (PDB ID: 4IBJ) and VPL60 (PDB ID: 4IBK). For EBOV-Z VP40, an energy cut-off (−6.0 kcal/mol) was set because there was no ligand-bound VP40 structure present in PDB (Table S1).

Based on virtual hits binding energies, we decided to select the binding energy cutoff value on both proteins. Particularly, based on Autodock Vina docking score (ΔG), 451 compounds with binding energies lower than −6.8 kcal/mol for EBOV-Z VP35 and 874 compounds with binding energies lower than −6.00 kcal/mol for EBOV-Z VP40 were selected. These virtual hits were filtered in terms of their DSX free energies of binding. Cut-off values lower than −95.82 kcal/mol for EBOV-Z VP35 and lower than −91.25 kcal/mol for EBOV-Z VP40 led us to consider 296 and 453 compounds, respectively. In order to further screen these 749 compounds, protein complexes were visually inspected and 91 compounds were finally selected with promising high binding energies against both EBOV-Z VP35 and VP40. These virtual hits were screened through extensive ADMET analyses and carefully analysed for molecular interactions with binding site residues.

### 2.3. Prediction of Pharmacokinetic Properties and Toxicity Assessment

Pharmacokinetic properties (PK) and toxicity depend on the molecular descriptors of the compound. The Molinspiration online server was used to check the phytochemicals as drug candidates based on Lipinski’s filter (log*p* ≤ 5, molecular weight ≤ 500, hydrogen bond acceptors ≤ 10, hydrogen bond donors ≤ 5). The bioavailability of these compounds was also determined by total polar surface area (TPSA) analysis, as this has been reported to correlate with excellent human intestinal absorption (HIA) and Caco-2 cell permeability. According to Veber’s rule for good oral bioavailability, the number of rotatable bonds must be no more than 10 while TPSA values must be ≤140 Å. %ABS were estimated from the predicted TPSA values. It has been reported that passively absorbed molecules with a TPSA greater than 140 Å will have low oral bioavailability. According to the standards mentioned above, estimated percentages of absorption for common compounds ranged from 54.12% to 88.53%. In silico predictions of PK properties such as absorption, distribution, metabolism, excretion, and toxicity (ADMET) are valuable tools to determine the likelihood of success of compounds for potential human therapeutic use [39]. The ADMET properties of 91 common compounds of EBOV-Z VP35 and VP40 proteins were analysed using admetSAR (Available online: http://www.admetexp.org). It is essential for a parental compound to have an impressive ADMET profile. The BBB [40], HIA [41], aqueous solubility [42], Caco-2 cell permeability, CYP 450 inhibition [43], and AMES toxicity were calculated for 91 compounds and 13 virtual hits were able to pass these series of ADMET filters, as summarised in Table 1.

On the basis of the admetSAR prediction, all compounds were able to penetrate the BBB, in addition to HIA, and were revealed as non-inhibitors for the P-gp inhibitor. None of the phytochemicals, with the exception of compound **9**, showed any inhibitory effects on the renal organic cation transporter (ROCT). CYP enzymes, including various CYP450 substrates and inhibitors, play a fundamental role in drug metabolism. The results showed that all compounds revealed low CYP inhibitory promiscuity because these compounds were non-inhibitors for most of CYP450 enzymes, i.e., 2C9, 2D6, 2C19 and 3A4. Further drug metabolism analyses showed that all common compounds (Table 1) were non-substrates for two CYP450 substrates (2C9, 2D6). Moreover, toxicity analyses based on AMES test data revealed that none of the compounds were toxic or carcinogenic.

Virtual hits were further filtered through a series of drug safety profiling parameters and checked for any undesirable moieties and substructures involved in potential toxicity through a series of PAINS (Pan Assay Interference Compounds) filters. Compounds **8**–**10**, **12** and **13** were not identified by PAINS-1, 2 and 3, passed all oral bioavailability and drug safety filters, and were classified as acceptable; compounds **1**–**7** and **11** were classed as intermediate and rejected. The latter compounds had undesirable substructural moieties (low- and high-risk coumarines) and displayed issues with drug safety profiling (Table 2).

### 2.4. Molecular Interaction with EBOV-Z VP35 and VP40

**Figure 4:**
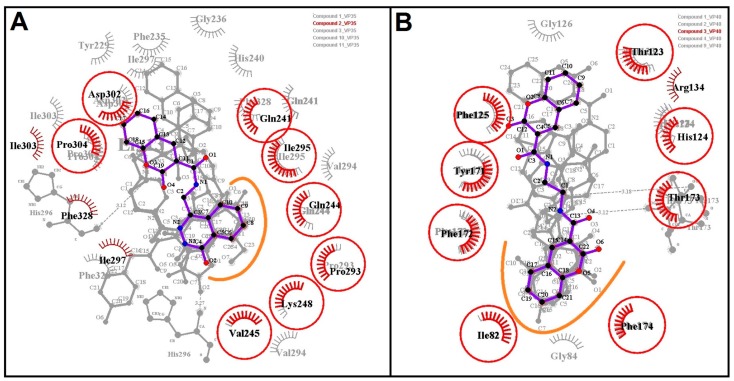
Protein ligand analysis by ligplot. Ligplots showed the conserved binding modes (outlined by the orange lines) of virtual hits with respective targets: EBOV-Z VP35 (**A**) and VP40 (**B**). Conserved interacting residues are displayed in red circles.

**Figure 5:**
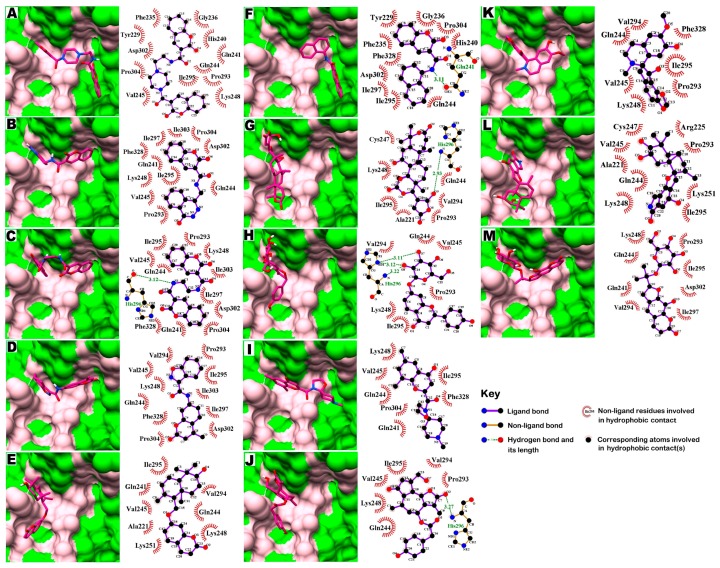
Molecular surface representation of EBOV-Z VP35 with respective ligands. (**A**–**M**) Molecular surface representation (in green) with respective ligands displayed in stick format (in magenta). Binding site residues are in pink. Alongside each 3D complex are schematic representations of the 2D interactions (with a cutoff distance of 4 Å) between each ligand and EBOV-Z VP35 amino acid residue using Ligplot analysis. Residues involved in hydrophobic contacts are black and demarcated by a spoked red arc, while those involved in hydrogen bonding are green, along with the values of the distances. Atoms are shown in white for carbon, red for oxygen, and blue for nitrogen.

**Figure 6:**
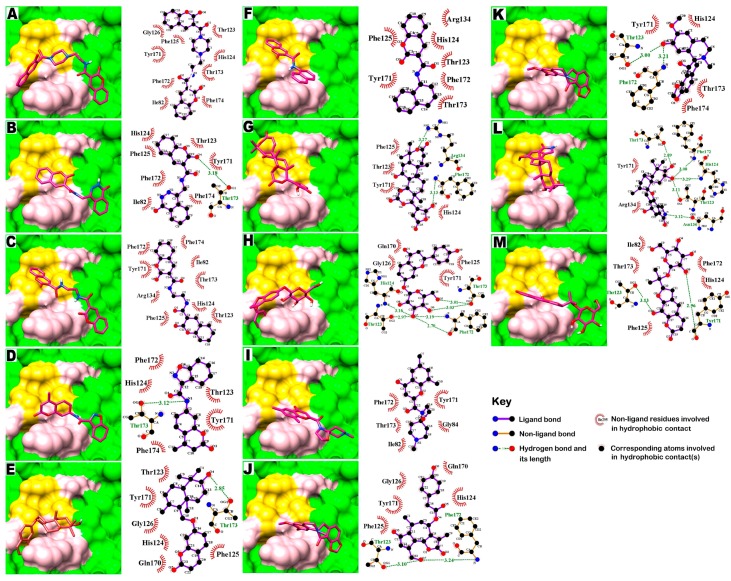
Molecular surface representation of EBOV-Z VP40 with respective ligands. (**A**–**M**) Molecular surface representation (in green) with respective ligands displayed in stick format (in magenta). Binding site residues are in pink. Residues Thr123, Phe125, and Arg134 form the catalytic triad in the active sites that interact with RNA are displayed in yellow. Alongside each 3D complex are schematic representations of the 2D interactions (with a cutoff distance of 4 Å) between each ligand and EBOV-Z VP40 amino acid residue using Ligplot analysis. Residues involved in hydrophobic contacts are black and demarcated by a spoked red arc, while those involved in hydrogen bonding are green, along with the values of the distances. Atoms are shown in black for carbon, red for oxygen, and blue for nitrogen.

Protein complexes of EBOV-Z VP35 and VP40 with 13 hits were critically inspected by post-docking analysis. All 13 compounds showed high binding affinities with EBOV-Z VP35 and VP40, ranging from −9.2 to −7.0 kcal/mol and −7.2 to −6.4 kcal/mol with Autodock Vina, and ranged from −156.867 to −95.53 kcal/mol and −120.189 to −91.619 kcal/mol, respectively, with the DSX rescoring function, as tabulated in Table 3. Ligplot analysis of both protein complexes inferred all-inclusive findings. Many virtual hits, when docked with both proteins, adopted the same orientation for their single bicyclic ring, highlighted by the curved line in Figure 4. These included compounds **1**–**3**, **10**, and **11** against VP35 and compounds **1**–**4** and **9** against VP40. All virtual hits also docked within the surface groove of IID near the FBP surrounded by binding site residues, as determined by binding site analysis, including Ile295, Gln241, Gln244, Pro293, Lys248, Leu249, Lys251, Ile297, Asp302 and Phe328. Lys251 is a critical residue for VP35 polymerase cofactor function, as mutations of Lys251 led to the loss of function [44]. With VP40, virtual hits were found interacting with three highly conserved RNA interacting residues, i.e., Thr123, Phe125, and Arg134 as highlighted yellow surface along with other core residues. Molecular docking simulations identified those important virtual hits that participated in hydrogen and hydrophobic interactions with many key residues of both proteins. In complexes with EBOV-Z VP35, compounds **3**, **6**–**8** and **10** showed at least one or more hydrogen bonds with O and N atoms of Gln241 and His296, sharing an H-bond distance between 2.93 to 3.27 Å (Figure 5C,F–H,J, Table 3). Whilst in EBOV-Z VP40, All compounds except **1**, **3**, **6** and **9** were involved in at least one or up to six H-bonds, having molecular distances between 2.7 and 3.29 Å (Figure 6B,D,E,G,H,J–M, Table 3). H-bond analysis of EBOV-Z VP40 complexes showed that the OG1 atoms of Thr123 and Thr173, and N atoms of Phe172 and His124, actively participated in H-bonds with many compounds. Most interestingly, compounds **8** and **12** contributed to a network of **6** and **5** H-bonds with important binding site residues. Besides H-bonds, a large number of hydrophobic interactions were present with the residues surrounding the IID of VP35 and RNA interacting site of VP40, as represented in Figure 5 and Figure 6. The 2D analysis further revealed that Ile295, Gln244, Lys248, Pro304, Phe328, Ile297 of VP35 and Tyr171, Phe172, Thr173, His124, Phe123 of VP40 could form a network of hydrophobic interactions. Consequently, this contributed to the binding energies of docked compounds as these interactions mediated the firm binding of compounds to the binding site of the respective protein, inhibiting its function. Table 3 also reports binding energies with corresponding DSX-scores. The rescored binding energies reported by DSX forms the total score, including possible torsional and intramolecular interactions. Along with Per Contact Score (PCS), the score is divided by the total number of atomic interactions that show any contribution to obtain the final score within 6 Å.

### 2.5. Alignments and Structural Studies

**Figure 7:**
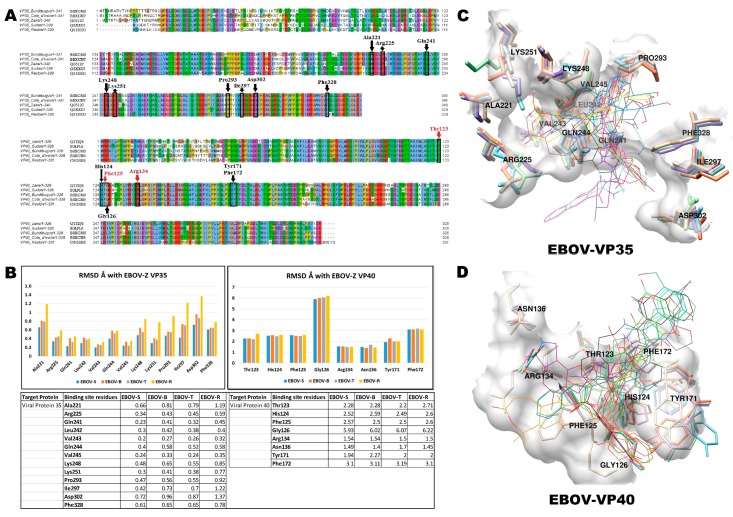
Structural analysis of different Ebola strains. (**A**) Multiple sequence alignment of viral protein 35 (VP35) and 40 (VP40) of Ebola *Zaire*, *Reston*, Côte d’Ivoire, *Sudan* and *Bundibugyo* along with their chain length. Residues are coloured according to default colouring scheme of clustalX. Important structural residues of binding sites of VP35 and VP40 among the five Ebola subtypes are highlighted with a black outline, and labelled by residue name, as indicated by an arrowhead. The red arrowhead indicates RNA interacting residues (Thr123, Phe125 and Arg134) of VP40, which are also conserved among all subtypes. Sequences of representative Ebola virus subtypes were retrieved from GenBank and aligned using ClustalW. The alignment was further hand curated using Jalview 2.7; (**B**) Structural comparisons of conserved binding site residues. Individual RMSD values of conserved binding site residues (VP35 in left, VP40 in right) of all Ebola strains are plotted against the corresponding residues of EBOV-Z. The RMSD analysis shows that binding site residues of EBOV-S, EBOV-R, EBOV-B and EBOV-T contain a close structural similarity with corresponding residues of EBOV-Z; Structural superimposition of VP35 (**C**) and VP40 (**D**) are displayed in the bottom. Amino acid side chains highlight the conserved binding site residues of VP35 and VP40 in all strains as EBOV-Z (tan), EBOV-S (plum), EBOV-T (green), EBOV-B (sky blue), EBOV-R (coral), top ligands (wire form) are docked in the binding site of EBOV-Z. Molecular surface of the EBOV-Z binding site is highlighted in brown.

Multiple sequence alignments of the EBOV-Z VP35 and VP40 protein sequences (Figure 7A) across five different strains showed extremely high conservation throughout the entire length of the sequences, including 193 residues in VP35 and 217 residues in VP40; these residues are completely conserved among all the Ebola strains examined. Residue conservation across the Ebola virus strains is suggestive of the importance and necessity of these residues in Ebola virus functions. This inferred a strong selection pressure to prevent any variation in sequence, and may also highlight important functional domains and structural features. This is evident from sequence alignment, in that the structurally important residues present in the binding site were conserved in EBOV VP35 and VP40 of all Ebola strains under investigation (Figure 7A). To determine the probable conserved binding site residues of Ebola strains, an effort was made to map the binding site residues of all strains by superimposing their 3D structures. In this regard, homology modelling of EBOV VP35 (*Sudan*, *Tai Forest* and *Bundibugyo*) and VP40 (*Tai Forest*, *Bundibugyo* and *Reston*) was performed. The homology-based search inferred that the 3D coordinate crystal structure of the *Reston* Ebola virus RNA binding domain (PDB ID: 3KS4), in addition to the crystal structure of the *Sudan* Ebola virus matrix protein VP40 (PDB ID: 3TCQ), were the best hits based on query coverage, E-value, and identity; therefore, this was considered to be the best template for homology modelling (Table 4). For both 3D structures, chain A at 3.0 Å was used as a coordinate structure for homology modelling. As presented in Figure 7B, all binding residues of VP35 in EBOV-S, EBOV-B, EBOV-R, and EBOV-T exhibited close structural similarities with EBOV-Z respective residues by sharing an RMSD (root mean square deviation) value of 0.2–1.5 Å, respectively. Similarly, in VP40, the superimposition of highly conserved RNA interacting residues Thr123, Phe125, and Asn134 of EBOV-S, EBOV-B, EBOV-R, and EBOV-T resulted in an RMSD value between 1.5 and 2.71 Å. The data revealed that the binding site residues of VP35 and VP40 of EBOV-S, EBOV-B, EBOV-R, and EBOV-T maintained a similar conformational pattern to that of EBOV-Z by sharing a common 3D structural arrangement (Figure 7B). To validate the biological significance of the binding site residues of all Ebola strains, 3D structural comparisons of VP35 and VP40 for EBOV-Z, EBOV-S, EBOV-B, EBOV-R and EBOV-T were performed (Figure 7C,D). These structures were superimposed to compare the conformations of conserved binding site residues structurally, in order to further narrow down the interaction crosstalk against docked complexes of EBOV-Z VP35 and VP40. To further increase the scope of underlying study, reported mutations were traced out in both proteins from the data published by Gire et al., of EBOLA surveillance. The study reported that Arg37, Ser41, Val170, Thr191, Asn254, Ile258, Ser272 and Ala9, Val20, Leu75, Ala77, Ile94, Pro131, Pro164, Val166, Thr183, Thr197, Ser278, Ile324 in EBOV-Z VP35 and VP40 carried unique bases in 2014 outbreak strains, while VP35-Val87, Ala156, Asp204, VP40-Ile94, and Thr65 were polymorphic across the 2014 outbreak isolates [45]. The presented data clearly accords with the conservation of binding site residues across all strains of EBOV.

### 2.6. Retrospective Evaluation Virtual Screening Method

**Figure 8:**
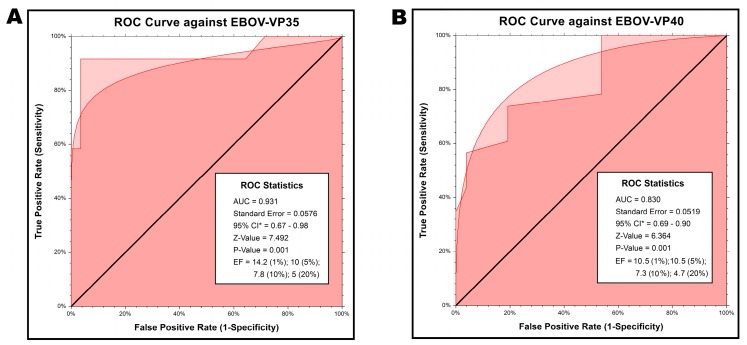
ROC curve analysis. ROC curve analysis of actives and inactives for detecting the performance of the VS method being employed, with the true- and false-positive rates in the *X*- and *Y*-axis, respectively; (**A**) The AUC of 0.931 (95% CI, 0.67–0.98) against EBOV-VP35; (**B**) the AUC of 0.830 (95% CI, 0.69–0.90) against EBOV-VP40. ROC, receiver operating characteristic, both by binomial and empirical approach; AUC, area under the ROC curve; CI, confidence interval; Z-value, Z-score for testing the designated hypothesis test; *p*-value, probability level associated with the Z-value; EF, Enrichment factor.

Retrospective validation is usually considered a benchmark for the success of VS methods. It enumerates the number of actives found with respect to the fraction of inactives. The NSCC.11 statistical package was employed to determine the ROC curve plotted between the true positive rate (Sensitivity) and the false positive rate (1–specificity) and the areas under the ROC-curves (AUC) were calculated for comparison. Conjointly, the enrichment factor (EF) was also calculated in terms of the ratio of true positives in the hit list at a given percentage of the database. These parameters provide a useful and practicable evaluation performance for determining the discriminatory power of the VS protocol [46]. The value of AUC fluctuated between 0 and 1, where 1 represents a perfect screen whilst 0.5 relates to a random screen. Figure 8 represents the overall profile of percentage of ligands found at ordinate (true positives) and at abscissa (false positives) positions for EBOV-VP35 (Figure 8A) and VP40 (Figure 8B). The ROC curve signified the evolution of sensitivity as well as specificity, and sensitivity as a function of (1–specificity).The percentage actives were also plotted against percentage inactives at all possible detected thresholds. The data using the ROC curve indicated a noticeable separation between the groups, with an AUC of 0.931 (Standard Error, 0.057) and a 95% CI (confidence interval) of 0.67 to 0.98 for EBOV-VP35 while the sensitivity and specificity were 80.77% and 76.91%, respectively. On the other hand, EBOV-VP40 showed a sensitivity and specificity of 76.36% and 71.11%, respectively, with an AUC of 0.830 (Standard Error, 0.0519) and a 95% CI (confidence interval) of 0.69 to 0.90. The enrichment formulation is also used to report the ratio of true positives (found on the *Y*-axis in an ROC plot) to false positives (the *X*-axis in an ROC plot). The EF was calculated at different false positive rates in the ranked database. Therefore the theoretical maximum EF for subset levels of 1%, 5%, 10% and 20% were 100, 20, 10 and 5, respectively. For EBOV VP35, 14.2%, 57.14%, 78.57% and 100% of the known actives were found in the top 1%, 5%, 10% and 20% of the docked ranked database, respectively, corresponding to EFs of 14.28, 10, 7.85 and 5. Corresponding EBOV VP40 values were10.5%, 52.6%, 73.6% and 94.7% of VP40 virtual hits, producing EFs of 10.52, 10.52, 7.36 and 4.73, respectively (Figure 8A,B).

### 2.7. Aggregator Advisor Screening

To determine whether a virtual hit is already known to aggregate, leading to non-specific inhibition, Aggregator Advisor was employed on 13 virtual hits. The Aggregator Advisor works in close collaboration with lipophilicity and similarity thresholds (Tonimoto coefficient, *T*c). If the calculated Log*p* was >3, five out of seven compounds with *T*c values ≥95% of known aggregators aggregated at relevant concentrations; 10 out of 19 compounds with *T*c values between 94% and 90%, and three out of seven compounds with *T*c values between 89% and 85% also aggregated. Another three compounds within these ranges were weak aggregators and may aggregate at higher concentrations. All 13 virtual hits were checked for aggregator potential, as presented earlier in Table 3. In the default affinity range of 0.1–10 µM, Aggregator Advisor indicated that seven compounds had not previously been reported as aggregators, or as having similarities to a known aggregator. With Log*p* < 3*T*c < 85%, compound **5** was predicted to be dissimilar to any known aggregator in the database, but this molecule had a relatively high calculated Log*p* of 5.3, which was in the range reported for many other aggregators, so appropriate controls need to be performed in vitro in order to test for possible aggregation. Compounds **2**–**4** were similar to compounds that have previously been reported as aggregators with similarities of 90%, 93% and 87% respectively [47].

### 2.8. Metabolic Sites Analysis

**Figure 9:**
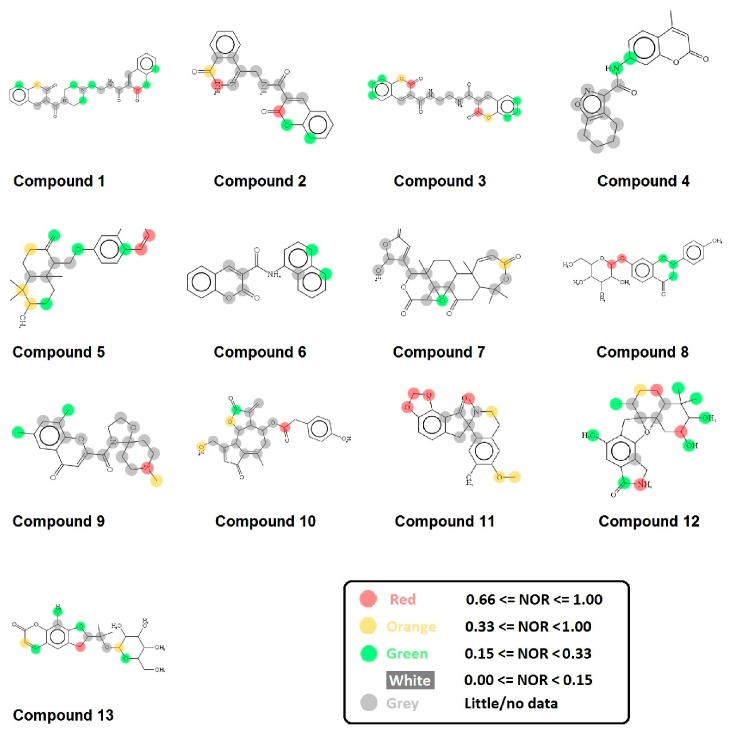
Metabolic sites prediction by MetaPrint2D. Diagrammatical representation of MetaPrint2D plots of common compounds (2-D format) for Ebola viral proteins, VP35 and VP40. Sites of metabolism are representing by colours as Red: most favourable site, Orange: medium/moderate, Green: Low, very low is uncoloured; and grey: no data available. NOR represents Normalized Occurrence Ratio; high NOR showed the more frequently reported metabolic sites in the metabolite database.

MetaPrint2D is a quick, productive, and precise predictor of metabolic sites and products of metabolism in small compounds, using circular fingerprints and substrate/product proportions. The atoms displayed in red, orange, green, and white represent the most favourable metabolic sites, followed by medium, low, and very low, respectively. In this current study, MetaPrint2D predicted that most of the virtual hits favoured metabolic sites, i.e., various methoxy, oxygen, and nitrogen groups of virtual hits, followed by red, orange, and green groups (Figure 9). As Figure 9 shows for compounds **1**, **2** and **5**, the most favoured and moderate metabolic sites were associated with oxygen and methoxy groups.

## 3. Discussion

Ebola VP35 plays a major role in viral assembly, as it acts as a viral assembly factor and also as a crucial component of the viral RNA polymerase complex. It impedes the host immune response by interfering with interferon (IFN) production. The dsRNA binding cluster, which is reported to be centred on Arg312, is highly conserved and fundamental for EBOV virulence [48]. Inhibiting VP35 activity causes reduced viral amplification and lethality in infected mice [49]. The EBOV VP35 is thus a vital drug target because of its multifunctional role in viral replication, the antagonising of host immune responses, and its role as a cofactor for the viral polymerase complex [28].

Ebola viral protein VP40 is reported to be associated with the assembly budding process and stability of the virus. It contains two short sequence motifs, i.e., PPXY and PTAP found at its N-terminus. It is implicated in virus release via interaction with cellular factors [18]. The N-terminal domain of VP40 is also involved in dimerization, whereas the C-terminal domain harbours membrane binding motifs [50]. Inhibition of VP40 results in failure of viral particle formation. Interestingly, in the absence of other proteins, VP40 continues to form virus-like particles when expressed in a human cell [51]. Furthermore, VP40 forms associations with microtubules and actin that help in movement and assembly [52]. Most importantly, it plays a crucial role in viral transcription by forming an RNA binding octameric ring. Both Ebola targets were considered as having the potential for screening large compound libraries in early drug discovery processes and several high-throughput studies have been performed recently on drug repurposing. However, to avoid the risk of rapid development of drug resistance, repurposed drugs must follow strict criteria because a few mutations can drastically alter the biological properties of RNA viruses [24,26,27,30,53,54,55,56].

The development of Ebola drugs is still remote from having FDA approval. Despite all experimental data, drugs that were thought could eliminate Ebola virus did not work. Production of an effective drug, ZMapp, for instance, is costly and time-consuming, and it was not subjected to clinical trials to check for its efficiency [10]. Lamivudine, being inexpensive, has been chosen as the best option for further analysis. The success rate of two new drugs, GS-5734 and BCX-4413, has been reported to be 100%. These drugs, however, have not as yet been tested in humans. Brincidofovir has a high success rate and has been tested in 1000 humans against many viruses except Ebola. Information about this drug’s effectiveness against Ebola is still lacking. Brincidofovir and T-705 have an advantage over other drugs as they both may be administered in the form of tablets, making distribution easier and thus increasing its potential effectiveness against Ebola virus. For these reasons, the T-705 trial has been approved to be expanded to examine this drug’s efficacy in a larger population of Ebola-infected people.

The current systematic study attempts to identify anti-EBOV compounds of plant origin that may be considered as parental compounds for antiviral drug development. Structure-based virtual screening of 145,329 natural compounds against two EBOV proteins has assisted in robust screenings for the novel, potent virtual hits. These (a) followed drug-likeness and Lipinski’s Ro5 as important criteria for characterising novel hits by screening large chemical libraries. Compounds violating more than one drug-like parameter may have issues with bioavailability and therefore were eliminated from the study; (b) showed successful BBB and HIA since polar molecules are poor CNS drugs, whereas moderately lipophilic compounds cross the BBB. Through the process of molecular docking, the best possible orientations forming stable ligand-target protein complexes were achieved, coupled with a Molecular Interaction Field (MIF) strategy based on rescoring function. Based on the experimentally determined VP35 inhibitors by Brown et al., the binding energy cutoff was set to select for possible dual virtual hits that could act on both EBOV proteins. By the application of a combined binding energy cutoff, 749 hits remained, which were further reduced to 91 that showed high binding affinity for both EBOV proteins. Recent crystallography analyses also recognised identical ligands for different proteins [57]. The present study revealed a varying trend in docking results obtained against both Ebola viral proteins (VP35 and VP40) of the *Zaire* strain. This was conducted using Autodock Vina alongside the rescoring function of DSX-score to evaluate binding energies. Based on a comparative assessment of scoring functions, as determined by Cheng et al., (2009) DSX devised by Kelbe achieves better results regarding docking power [58]. Further, CSD-based potentials yield better results compared to PDB-based potentials due to the availability of better resolved small crystal structures and more comprehensive contact data for a specific set of atoms. The structures of several compounds in complexes with VP35 and VP40 highlighted several H-bonds and hydrophobic interactions between functional groups, and side chains of essential residues for Ebola viral protein functions. High binding energies (−9.2 to −7.4 kcal/mol) were observed against VP35, whereas the binding energy for VP40 ranged from −7.2 to −6.4 kcal/mol. The efficiency of ligand–protein complex formation can be quantified by calculating the binding energy of the ligand and its half-life. With an increase in the value of binding energy, the rate of dissociation slows. For weaker interactions, the rate of dissociation is rapid [59]. This suggests that compounds with strong binding energies take a longer time to dissociate and thus have a longer half-life. Strong interactions between residues imply that binding with inhibitory compounds may be stable, leading to an inhibitory reaction. Interestingly, binding orientations of top hits with interacting residues of VP35, as evident from this investigation, was also demonstrated through co-crystallized structures of specific VP35 inhibitors [53] (Figure S1). To predict the conformation of compounds on other Ebola strains, binding sites were superimposed, providing a strong indication of multi-targeted behaviours of the virtual hits.

Interestingly, all top hits showed promising ADMET properties; the body can either metabolise poor PK/PD candidates, might result in toxicity or be unable to cross membranes. Furthermore, the cytochrome P450 analysis was carried out for its most crucial isoforms: CYP1A2, CYP2A6, CYP2C9, CYP2C19, CYP2D6, CYP2E1 and CYP3A4. The cytochrome P450 superfamily plays vital role in drug metabolism and excretion from the liver [48]. Inhibition of these isoforms encourages drug interactions, due to which, a co-administered drug may fail to metabolise and can accumulate in the body to toxic levels. The Log S or aqueous solubility of a drug affects its absorption and distribution. Predicted solubilities of the virtual hits under study were within an acceptable range. Virtual hits were further checked for the presence of any toxicophore through a PAINS filter, oral bioavailability and drug safety profiling. According to Baell et al., PAINS moieties are compounds that appear as frequent hitters (as promiscuous compounds) in a number of biochemical high throughput screens [60]. Consequently, such compounds should be removed to prevent possible toxicity during early stages of drug development [61]. Limonin [62] and Neoglucobrassicin [63] were predicted as anti-Ebola compounds but showed high-risk structural alerts, including (a) high-risk epoxides that form protein adducts, thus potentially disturbing signal transduction cascades [64] and (b) high-risk quinones that lead to the formation of reactive oxygen species (ROS) and cause severe oxidative stress [65]. Furthermore, quinone-like compounds comprise structures that are widely reported to yield false positives or are inactive [60]. We, therefore, employed PAINS filters to analyse high-risk chemical groups, with the result that 5/13 virtual hits did not encounter any PAINS moieties and were therefore classed as acceptable.

Docking-based VS methods have been evaluated by exploring their ability to prioritize (i.e., rank) known active compounds that have been seeded into a collection of inactive (either known or presumed) compounds. In VS validation, success is defined as the ability to enrich some relatively small fraction of best-scored ligands with respect to the proportion of seeded known actives. Here we were interested in identifying a significantly larger fraction of true actives from a ranked database than from a random selection of compounds. Enrichment by VS methods may have a significant impact [66]. Therefore, enrichments were reported at different FP rates in Figure 8A,B. Notably, for subsets of 10% and 20%, the proposed VS method produced best enrichment factors of 7.85 and 5.45 for EBOV VP35, and 7.36 and 4.73, respectively, for EBOV VP40. Although early enrichment at 1% and 5% was a bit lower for both targets, this might be due to the low number of compounds in the ranked database. Eventually, the VS method showed the best enrichment at a 10% false positive rate. As every active compound in the ranked database raises the curve one unit on the *Y*-axis, a steep slope at the beginning of ROC thus indicates a successful early enrichment of actives amongst the uppermost ranked compounds.

A key challenge in target-based assays that have been specifically prominent in high throughput VS is the occurrence of a high rate of false positive hits (up to 95%) in a screen. These hits are likely non-specific compounds and are found because of assay artefacts such as aggregation. When Gossypentin and Taxifolin were screened as active multitarget inhibitors, these compounds were found to be 94% and 91%, respectively, similar to compounds that have been reported as aggregators [47,67,68,69,70]. Being a predominant mechanism for artefactual inhibition of proteins through non-specific interactions, several controls against this are now widely employed to screen for virtual hits with previously reported aggregators [71]. Therefore, we carefully analysed our best virtual hits for non-specific inhibition of aggregation in biochemical assays within the affinity range between 0.1 and 1.0 µM. Of the 13 virtual hits, six compounds showed similarities with reported aggregators by log*p* values and the Tonimoto coefficient, when analysed through Aggregator Advisor.

In summary, modern computer-based approaches are now becoming an important part of early drug discovery processes; consequently, this may result in the development of promising and efficient antiviral strategies. The Ebola virus is a worldwide threat, being extremely virulent and highly transmissible. For a drug to be successful against EBOV, it is important that it targets more than one protein and also different subtypes of the Ebola virus. It is possible to target more than one subtype if potential sites are conserved and do not affect the tertiary structure of the virus. We have analysed sequence variations through multiple sequence alignments between different subtypes of Ebola, where there was no substantial change in the secondary structure of the proteins, and the function or virulence of the virus was conserved. Many of the compounds studied here have not been tested for antiviral activities. We have highlighted dual virtual hits that have the potential to inhibit VP35 and VP40 proteins of the *Zaire* Ebola strain. Moreover, these compounds can be utilised as multi-target drugs against both viral proteins and other strains of the Ebola virus; Ebola *Zaire*, *Sudan*, *Reston*, *Bundibugyo* and Côte d’Ivoire. Extensive ADMET, drug safety profiling, and metabolic site analyses of virtual hits were carried out followed by PAINS filter and analysis of aggregator potential. Among the accepted virtual hits, four compounds had neither aggregator potential nor PAINS filter activity. Furthermore, retrospective validation via ROC curve confirmed our virtual screening workflow. Overall, we have used a systematic strategy to combine all of the above structural findings with available and modelled 3D structural information of Ebola VP35 and VP40 proteins. This study therefore provides a platform for the pharmaceutical industry and drug design laboratories to test virtual hits in vivo with the aim of developing successful drugs.

## 4. Materials and Methods

### 4.1. Dataset Preparation and Filtering Procedure

An exhaustive literature survey was performed for a collection of phytochemical libraries from e-molecule databases and natural product chemical companies including: Drug-like green collection of OTAVA chemicals (Kiev, Ukraine) (129,000), PubChem (Phytochemical compounds) (2845), Analyticon Discovery (The natural product company, Potsdam, Germany) (4967), Timtec LLC (Newark, DE, USA) (4553), Indofine Chemical company (The flavonoid company, Hillsborough Township, NJ, USA) (3833), and Selleck natural product library (131). All information on natural compounds was merged, thus obtaining an overall database of 145,329 natural compounds. These compounds included a wide range of chemical classes including flavonoids, terpenoids, poluines, lignans, polyphenolics, saponins, thiophenes, furyl compounds, alkaloids, coumarins, sulphides, polysaccharides, lectins, small peptides, and others. Duplicated structures were removed to acquire new scaffolds through InChlKey generated by Open babel [72]. Lipinski violations were calculated to check for oral bioavailability and pharmacokinetic parameters for crossing the blood–brain barrier (BBB), low toxicity with good solubility, and better human intestinal absorption (HIA). Compounds displaying these criteria were selected for further study. For retrospective validation of the database, actives and inactives against Ebola viral proteins, and decoys (compounds that are physicochemically similar to the active compounds apart from activity) were retrieved from PubChem and substance data were downloaded [73]. The 2D files of structures were converted to MOL 3D structures using Open babel and saved as .mol2 files [72]. Ligands were prepared by energy minimization using the Discovery Studio program 3.5, as designed by Accelrys Inc. (San Diego, CA, USA). Crystal structures of ZEBOV VP40 (PDB ID: 1H2C) [18], with a resolution of 1.60, and ZEBOV VP35 (PDB ID: 4IBK) [40] (Brown et al., 2014), with a resolution of 1.85, were obtained from the Protein Data Bank (Available online: http://www.rcsb.org/pdb/) [74] and visualized on Discovery Studio 3.5. Co-crystallized water molecules and small molecules were deleted to prepare the protein structure for multi-drug analysis. Energy was minimized for the 1000 steepest descent steps at a root means square gradient of 0.02, an update interval of 10 and with an AMBER ff12SB force field using UCSF Chimera 10.1 (Resource for Biocomputing, Visualization and Informatics, University of California, San Francisco, CA, USA) [75].

### 4.2. Evaluation of Protein Binding Site

Potential drug sites for VP35 and VP40 have previously been identified and experimentally confirmed in high-resolution crystals. Binding pockets of both proteins were examined from crystal structures and were further evaluated using the Castp server (Computed Atlas of Surface Topography of Proteins) (Available online: http://cast.engr.uic.edu), which locates all likely binding pockets. The Castp algorithm critically determines the area and volume of each binding pocket and possible cavities in a solvent accessible surface area [76].

### 4.3. Alignments and Homology Modelling

Multiple sequence alignment of VP35 and VP40 proteins of EBOV-Z, EBOV-R, EBOV-T, EBOV-S and EBOV-B were created using MUSCLE, version 3.7 [77] and checked visually using Jalview 2.7 [75]. Crystal structures of VP35 and VP40 of all Ebola strains were not present in the protein data bank repository. Therefore, to perform structural inferences, homology modelling was carried out for VP35 of EBOV-S, EBOV-T, and EBOV-B, and also for VP40 of EBOV-T, EBOV-B and EBOV-R. The protein sequences of all viral proteins were taken from UniProt and searched via a position-specific iterative BLAST (PSI-BLAST) [78] against the Protein Data Bank repository for a suitable template to generate a 3D coordinate structure. Initial alignment between target and template was generated using the ALIGN2D module. Then, the 3D coordinate structure of each viral protein was generated using a restrained-based approach in MODELLER.v9.12 [79]. MODELLER infers distance and dihedral edge restrictions on the target sequence from its alignment with 3-D template structures. These connections are communicated as contingent likelihood thickness capacities. Spatial limitations are inferred accordingly, with the stereochemistry authorized by CHARMM22 [80]. The power field terms were consolidated into an objective function that was minimized by an improvement method during model building. The predicted structures were refined using Procheck and structural alignment was performed to check conformations of binding site residues of VP35 and VP40 between all Ebola strains and respective RMSDs were calculated.

### 4.4. Structure-Based Virtual Screening

Autodock Vina, automated by Mcule drug discovery pipeline [81], was used to screen a natural compounds database [82]. The Mcule docking engine was efficient and used a gradient optimization method in its local optimization procedure. The gradient calculation algorithm effectively optimizes a sense of direction from a single evaluation [83]. For structure-based virtual screening (SBVS), only the EBOV-Z strain was considered because of the availability of well-resolved VP35 and VP40 crystal structures in the Protein data bank repository. Each ligand was docked into the respective binding sites of VP35 and VP40 of the EBOV-Z strain and was ranked as an energy function. The energy function consisted of protein and ligand van der Waals’ and electrostatic interactions [84].

Grids were created from a grid generation panel and relaxed on the investigated binding side of EBOV proteins. For VP35, a grid was made with dimensions of 30 Å × 30 Å × 30 Å, covering the IID First Batch Pocket (FBP); this was due to a previous investigation that found several small compounds that were capable of binding VP35 IID with high affinity and specificity [53]. For VP40, the interaction details of VP40 and RNA were taken from PDB and a 5 Å radius was selected for docking with selected phytochemicals. The grid was constructed with dimensions necessary to contain the RNA-interacting residues Thr123, Phe125 and Arg134. The protein complexes were analysed through PyMol (The PyMOL Molecular Graphics System, Version 1.5.0.4, Schrodinger, LLC) [85] and UCSF Chimera 10.1 (Resource for Biocomputing, Visualization and Informatics, University of California, San Francisco, CA, USA).

Further assessment of docking results was conducted employing DrugScore eXtended (DSX) [58] which is a program that analyses the output of Autodock. DSX uses a knowledge-based scoring function to evaluate binding energies of ligands bound to the delta-opioid binding site. The DSX-score uses statistical pair potentials derived from the Cambridge Structural Database (CSD) and PDB [86]. Moreover, the solvent accessible surface potential (SAS-potential) that is associated with PDB potential is introduced in a DSX-score that estimates desolvation effects. For the present work, CSD and SAR potentials were used. Ligands with a larger, negative DSX-score have an estimated higher binding energy.

### 4.5. Retrospective Virtual Screening Analysis

Retrospective validation of the capability of VS workflow to discriminate active compounds from inactives is a most reliable method if the test library covers known actives and experimentally verified inactive “True Decoy” compounds. Therefore, separation of actives in a pool of decoys was imperative [87,88,89]. Nevertheless, there is a dearth of data available for confirmed inactive compounds in public databases. We are grateful to Michael Lee for providing us with experimentally confirmed actives and inactives of EBOV-VP35, while for EBOV-VP40, there was not a single study that confirmed actives and inactives. Therefore, virtual hits against EBOV-Z VP40 were retrieved from the previously published in-silico studies including current study. In this regard, DUD-e (DUD, available online: http://dud.docking.org/) was employed to generate decoys for EBOV-Z VP40, and topological dissimilarities between decoys and virtual hits were calculated using Daylight fingerprints (Clustering package Irvine CA: Daylight Chemical Information System). Physical properties of virtual hits were taken in account to generate decoys possessing similar properties. For the assessment of virtual screening, a database was prepared consisting of experimentally confirmed 14 actives and 13 inactives against EBOV-VP35, whilst for EBOV-Z VP40, another database of 19 virtual hits and 50 decoys for each virtual hit were generated using DUD-e decoy generator, which were reduced to 44 on the basis of their molecular descriptors, leading to 836 decoys. To avoid biasing virtual screening results, we also generated set of 359 decoys with similar physicochemical properties to known actives of EBOV-VP35.These decoy sets were also seeded into corresponding databases. In such retrospective calculations, docked actives and inactives were ranked by score, and Area under Curve (AUC) was calculated by Receiver Operating Characteristics (ROC) curves and its 95% confidence interval was calculated using NSCC.11 statistical software (NCSS 11 Statistical Software (2016). NCSS, LLC. Kaysville, UT, USA) [90,91,92,93]. Early enrichment (EF) in true positives was computed at false positive rates by the following formula:

(1) $$EF=\frac{a/n}{A/N}$$

where *n* = a total number of hits, *a* = the total number of actives in the n hits, N = total number of compounds in the database, and A = the total number of actives in the database. Here we were more interested in enrichment factors (EF*_x_*) at 1% (EF_1_), 5% (EF_5_), 10% (EF_10_) and 20% (EF_20_) false positive rates (FP) in the ranked database of EBOV VP35 and VP40 as follows:

(2) $$EF_{x}=\frac{TP}{FP_{x}}$$

All compounds for retrospective validation were transformed to 3-D format and minimised using the procedure above.

### 4.6. Calculations of Pharmacokinetics and Toxicity Analysis

Physicochemical molecular descriptors and drug likeliness of phytochemicals were examined using a Molinspiration server (Available online: http://www.molinspiration.com) based on the Lipinski Rule of Five (LRo5) [91]. Compounds containing a violation according to the LRo5 were removed from further analysis. The Molinspiration server predicts the most important properties of compounds such as Log*p*, molecular weight, hydrogen bond acceptors and donors, and total polar surface area (TPSA). The percentage of absorption (%ABS) was measured from the equation: %*ABS* = 109 − (0.345 × *TPSA*) [92]. In order to identify possible adverse effects of virtual hits in humans, the ADMET properties of the filtered compounds were inferred using a variety of tools. These included: the OSIRIS property explorer (Available online: www.organic-chemistry.org/prog/peo/), which is designed to highlight undesired effects, ADMET prediction suite by Advanced Chemistry Development (ACD/Labs) Software for rapid profiling and screening of compounds utilizing ADMET parameters, Molsoft (Available online: http://molsoft.com/mprop/) for calculation of drug-likeness properties and AdmetSAR (Available online: http://lmmd.ecust.edu.cn:8000/) for prediction of QSAR-based ADMET properties. This provides the user with a friendly interface for the latest and most comprehensive, manually curated database for a diverse range of chemicals linked with Absorption, Distribution, Metabolism, Excretion and Toxicity profiles [93]. Additionally, undesirable substructure moieties and the drug safety profile of all filtered hits were projected [60,94].

### 4.7. Aggregator Advisor Prediction

Colloidal aggregation of organic molecules is the predominant mechanism that leads to nonspecific inhibition [95,96] and sometimes activation [97,98]. This is a major source of false positive results in early drug discovery processes through virtual screening [37]. In order to predict final virtual hits that aggregate or may aggregate under biochemical assay conditions, Aggregator Advisor (Available online: http://advisor.bkslab.org/), a tool to predict aggregating compounds and to advise on the likelihood of aggregation, was employed. This is based on chemical similarities to known aggregators (Tonimoto coefficient), and physical properties (e.g., Log*p*) [99].

### 4.8. Metabolic Site Prediction

MetaPrint2D (Available online: http://www-metaprint2d.ch.cam.ac.uk/) is a web-based tool that predicts metabolic sites of compounds that undergo phase 1 metabolism based on their similarities to known metabolic sites. MetaPrint2D predicts the metabolism of xenobiotics through data mining and statistical analysis of known metabolic transformations, as reported in the scientific literature [100]. This user-friendly software predicts the metabolic site by uploading a SMILES (Simplified molecular-input line-entry system) format of compounds.

## 5. Conclusions

Involvement of computational tools has been widely used for drug development and discovery. Viral target identification and investigation of host proteins involved in causing Ebola virus are currently in focus. Lack of information regarding viral mechanism of action, binding sites and Ebola drug targets poses a challenge in regard to the discovery of EBOV inhibitors. The design of effective inhibitors with a potential to be used as anti-Ebola drugs can help to combat this disease.

## Figures and Tables

**Table 1:** Predicted ADMET assessment of 13 virtual hits.

ADMET	Compd. 1	Compd. 2	Compd. 3	Compd. 4	Compd. 5	Compd. 6	Compd. 7	Compd. 8	Compd. 9	Compd. 10	Compd. 11	Compd. 12	Compd. 13
BBB	+	+	+	+	+	+	+	+	+	+	+	+	+
HIA	+	+	+	+	+	+	+	+	+	+	+	+	+
Caco-2 permeable	−	_	−	−	+	+	−	−	+	+	+	−	−
Solubility	−3.16	−3.1	−3.25	−3.25	−5.24	−4.33	−4.38	−2.44	−2.53	−2.89	−2.62	−3.63	−2.53
P-gp													
Substrate	+	−	−	+	+	−	+	−	−	−	+	−	−
Inhibitor	−	−	−	−	+	−	+	−	−	−	−	−	−
ROCT	−	−	−	−	−	−	−	−		−	+	−	−
CYP450 substrate													
2C9	−	−	−	−	−	−	−	−	−	−	−	−	−
2D6	−	−	−	−	−	−	−	−	−	−	+	−	−
3A4	−	−	−	−	+	−		−	−	−	+	+	+
CYP450 inhibitor													
1A2		+	+	+	+	+	−	−	−	−	−	−	−
2C9	−	−	−	−	+	−	−	−	−	−	−	−	−
2D6	−	−	−	−	−	−	−	−	−	−	−	−	−
2C19	+	−	−	−	+	−	−	−	−	−	−	−	−
3A4	−	−	+	−	+	−	−	−	−	−	+	−	−
CYP IP	Low	Low	Low	Low	Low	Low	Low	Low	Low	Low	Low	Low	Low
AMES toxicity	−	−	−	−	−	−	−	−	−	−	−	−	−
Carcinogens	−	−	−	−	−	−	−	−	−	−	−	−	−

ADME, absorption distribution metabolism elimination; BBB, blood–brain barrier; HIA, human intestinal absorption; CYP450, cytochrome P450; CYP IP, CYP inhibitory promiscuity; ROCT, renal organic cation transportation; +, present; −, not present.

**Table 2:** Screening of 13 virtual hits from series of filters being applied.

Virtual Hits	Oral Bioavailability				ADMET	Drug Safety Profiling			Filtered State	Undesirable Structuresmioties	Aggregator Advisor		
	Drug Likeness	Lipinski’s Ro5	Veber Rule	Egan Rule		GSK 4/400 Rule	Pfizer 3/75 Rule	Lilly MedChem Rules			Aggregator Likelihood		Similar with
**1**	√	√	√	√	√	√	×	√	Rejected	High_risk coumarines	Reported as an aggregator		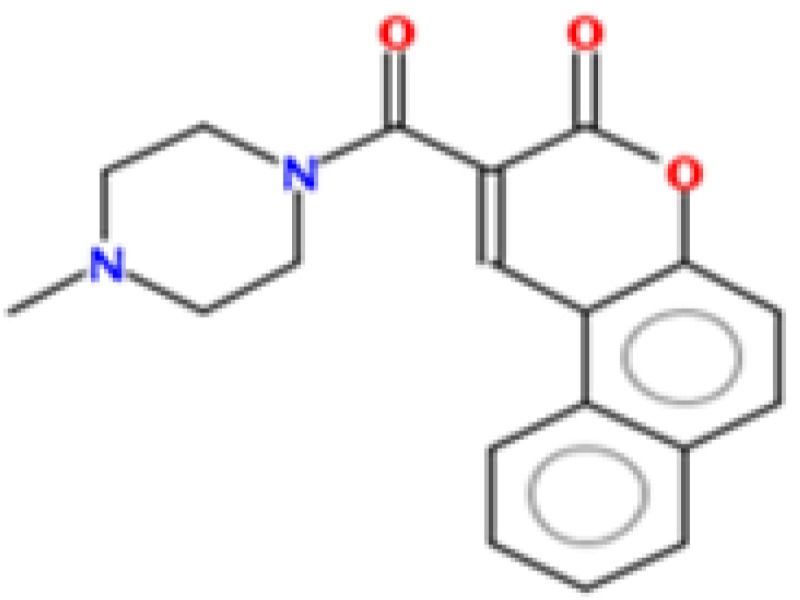
											Log*p*: 1.6	*T*c: 97%	
**2**	√	√	√	√	√	√	√	√	Intermediate	Low_risk coumarines	Non-aggregator Log*p*: 2.0		
**3**	√	√	√	√	√	√	×	√	Rejected	High_risk coumarines	Reported as an aggregator		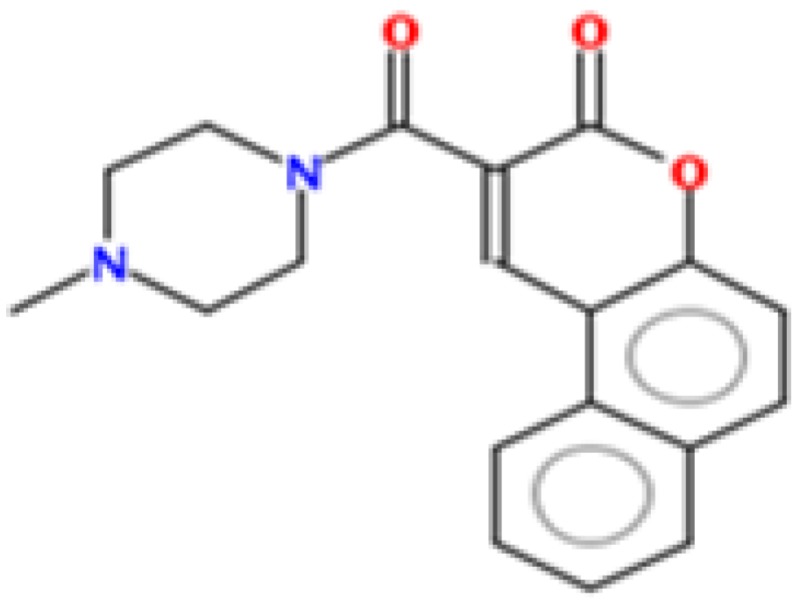
											Log*p*: 2.0	*T*c: 93%	
**4**	√	√	√	√	√	√	√	×	Intermediate	Low_risk coumarines	Reported as an aggregator		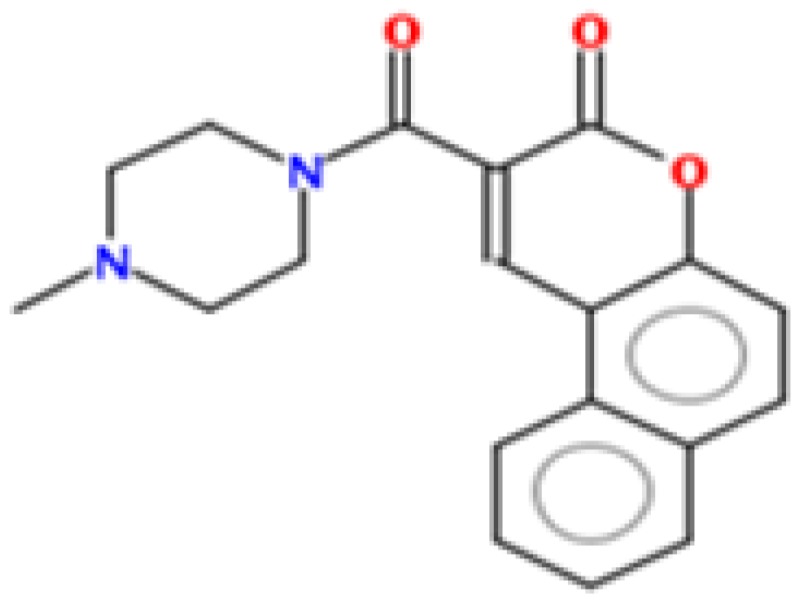
											Log*p*: 3.2	*T*c: 76%	
**5**	√	√	√	√	√	√	×	√	Intermediate	Low_risk coumarines	Not similar to any known aggregator in in-house database High Log*p*: 5.2		
**6**	√	√	√	√	√	√	×	×	Intermediate	Low_risk coumarines	Reported as an aggregator		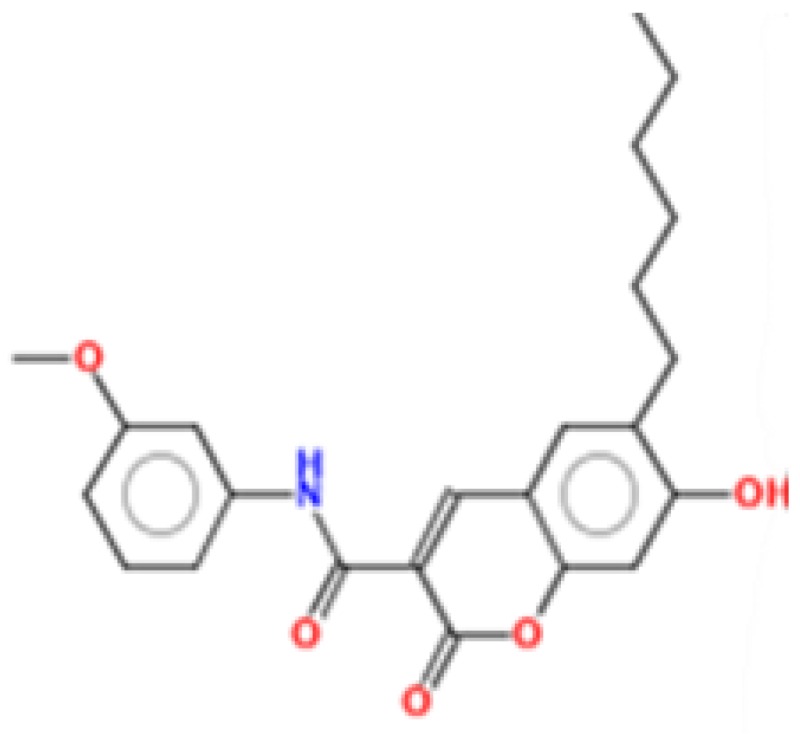
											Log*p*: 4.0	*T*c: 75%	
**7**	√	√	√	√	√	√	√	×	Rejected	High_risk epoxide	Non-aggregator Log*p*: 2.8		
**8**	√	√	√	√	√	√	√	√	Accepted	No	Non-aggregator Log*p*: 0.4		
**9**	√	√	√	√	√	√	√	√	Accepted	No	Non-aggregator Log*p*: 2.6		
**10**	√	√	√	√	√	√	√	√	Accepted	No	Non-aggregator Log*p*: 1.1		
**11**	√	√	√	√	√	√	×	√	Rejected	Frequent_hitter dopamine; Low_risk benzodioxolane	Non-aggregator Log*p*: 2.3		
**12**	√	√	√	√	√	√	√	√	Accepted	No	Non-aggregator Log*p*: 2.8		
**13**	√	√	√	√	√	√	√	√	Accepted	No	Reported as an aggregator		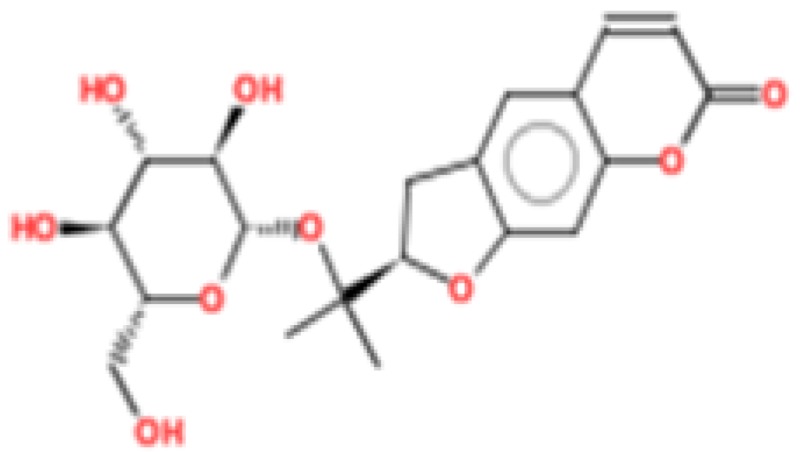
											Log*p*: 0.2	*T*c: 90%	

Veber Rule; Bad or Good oral bioavailability rule (rotatable bonds ≤ 10) and (TPSA ≤ 140 Å or H-Bonds Donors + H-Bonds Acceptors ≤ 12), Egan Rule; Bad or Good oral bioavailability rule (0 ≥ TPSA ≤ 132) and (−1 ≥ log*p* ≤ 6), GSK4/400 rule; Bad or Good ADMET profile (log*p* ≤ 4) and (Molecular Weight ≤ 400), Pfizer 3/75 rule; Rule which narrates to compounds with a log*p* (>3) and TPSA (<75) are almost 2.5 times more likely to be toxic as to be clean, Lilly MedChem Rules; a set of 275 rules to identify compounds that may interfere with biological assays in terms of reactivity, interference with assay measurements, activities that damage proteins, and lack of druggability, √; Compounds fulfilled the criteria, ×; couldn’t pass the criteria, Accepted; Compounds with no structural cautions, Intermediate; Compounds with low-risk structural cautions, Rejected; Compounds that include high-risk structural cautions, *T*c; Tonimoto coefficient.

**Table 3:** Molecular docking analysis.

	Source-ID/Name/Formula	Autodock Vina (ΔG)		DSX Drugscore				Molecular Interactions							
		EBOV-Z VP35 (kcal/mol)	EBOV-Z VP40 (kcal/mol)	EBOV-Z VP35		EBOV-Z VP40		EBOV-Z VP35				EBOV-Z VP40			
				Score (kcal/mol)	PCS	Score (kcal/mol)	PCS	Binding Site Interacting Residues	No. of H-Bond Interactions	No. of Hydrophobic Bonds Interactions	Total Number of Bonds	Binding Site Interacting Residues	No. of H-Bond Interactions	No. of Hydrophobic Bonds Interactions	Total number of Bonds
**1**	Timtec-ST45161107 2-Oxo-*N*-(2-{4-((2-oxo-2*H*-chromen-3-yl)carbonyl)-1-piperazinyl}ethyl)-2*H*-chromene-3-carboxamide C_26_H_23_N_3_O_6_	−9.2	−7.1	−156.867	−0.265	−120.189	−0.255	Lys248, Pro293, Ile295, Val245, Phe328, Ile297, Asp302, Pro304, Gln244, Tyr229, Gly236, His240, Gln241, Phe235	0	31	31	Gly126, Thr123, His124, Thr173, Phe174, Ile82, Phe172, Tyr171, Phe125, Gly126	0	30	30
**2**	Otava-7118230235 2-Oxo-*N*-((4-oxo-3,4-dihydro-1-phthalazinyl)methyl)-2*H*-chromene-3-carboxamide C_19_H_13_N_3_O_4_	−8.6	−6.9	−133.08	−0.291	−95.698	−0.229	Ile303, Pro304, Asp302, Gln244, Val245, Gln241, Phe328, Ile297, Lys248, Pro293, Ile295	0	27	27	His124, Phe125, Thr123, Tyr171, Thr173, Phe174, Ile82, Phe172	1 (3.18A) Thr173	30	31
**3**	Timtec-ST50912611 *N*,*N'*-1,2-Ethanediylbis (2-oxo-2*H*-chromene-3-carboxamide) C_22_H_16_N_2_O_6_	−8.2	−7.2	−152.793	−0.317	−115.494	−0.247	Val245, Pro293, Ile303, Ile295, Lys248, Gln241, Pro304, Gln244, Asp302, Phe328, Ile297	1 (3.12) His296	28	29	Phe174, Ile82, Thr173, His124, Thr123, Phe125, Arg134, Tyr171, Phe172	0	29	29
**4**	Timtec-ST50616170 *N-*(4-Methyl-2-oxo-2*H*-chromen-7-yl)-4,5,6,7-tetrahydro-1,2-benzoxazole-3-carboxamide C_18_H_16_N_2_O_4_	−8.3	−6.4	−111.212	−0.24	−93.831	−0.203	Val245, Pro293, Ile303, Ile295, Lys248, Gln241, Pro304, Gln244, Asp302, Phe328, Ile297, Val294	0	29	29	Phe174, Thr173, His124, Thr123, Phe125, Tyr171, Phe172	1 (3.12A) Thr173	28	29
**5**	Analyticon-NP-010155 7-{((1*R*,4a*S*,6*R*,8a*R*)-6-hydroxy-5,5,8a-trimethyl-2-methylidenedecahydronaphthalen-1-yl)methoxy}-2*H*-chromen-2-one C_24_H_30_O_4_	−8	−6.3	−105.282	−0.25	−98.943	−0.232	Val294, Ile295, Pro304, Gln241, Gln244, Lys248, Val245, Ala221, Lys251, Pro293	0	26	26	Thr123, Thr173, Phe125, His124, Gln170, Gly126, Tyr171	1 (2.85A) Thr173	23	24
**6**	Otava-0115540195 *N*-(1-Naphthyl)-2-oxo-2*H*-chromene-3-carboxamide C_20_H_13_NO_3_	−8	−6.9	−124.21	−0.269	−91.619	−0.283	Ile295, Ile297, Asp302, Phe328, Phe235, Tyr229, Gly236, Pro304, His240, Gln241, Pro293, Val294, Lys248, Val245, Gln244	1 (3.11) Gln241	28	28	Phe125, Arg134, His124, Thr123, Phe172, Thr173, Tyr171	0	20	20
**7**	Analyticon-NP-019744 Kihadarnin A C_26_H_30_O_9_	−7.8	−7.3	−102.276	−0.198	−91.924	−0.213	Gln244, Lys248, Ile295, Pro293, Leu249, Val294, Val245, His296	1 (2.93) His296	34	34	Phe125, Arg134, His124, Thr123, Phe172, Tyr171	1 (3.27) Arg134 1 (3.13) Phe172	21	23
**8**	Analyticon-NP-005474 2-(4-Hydroxyphenyl)-4-oxo-3,4-dihydro-2*H*-chromen-7-yl β-d-glucopyranoside C_21_H_22_O_9_	−7.7	−7	−123.944	−0.253	−116.708	−0.23	Val245, Lys248, Gln244, Ile295, Pro293, Val294, His296	3 (3.11A, 3.12, 3.22A) His296	26	29	Phe172, Thr123, His124, Gln170, Gly126, Phe125, Tyr171, Thr173	2 (3.01A, 3.03A) Thr173 2 (3.19A, 2.70A) Phe172 1 (2.97A) Thr123 1 (3.16A) His124	33	39
**9**	PubChem-CID17597017 6,8-Dimethyl-2-((8-methyl-1-oxa-4,8-diazaspiro(4.5)dec-4-yl) carbonyl)-4*H*-chromen-4-one C_20_H_24_N_2_O_4_	−7.6	−6.7	−98.897	−0.258	−91.398	−0.213	Pro293, Ile295, Phe328, Pro304, Gln244, Val245, Lys248, Val294	0	19	19	Phe172, Thr123, Phe125, Tyr171, Thr173	0	19	19
**10**	Analyticon-NP-000375 Lactupicrin C_23_H_22_O_7_	−7.3	−7.5	−95.53	−0.214	−116.548	−0.217	Val 294, Pro 293, Gln244, Lys248, Val245, Ile295, Val294	1 (3.27) His296	29	30	Phe172, Thr123, His124, Gln170, Gly126, Phe125, Tyr171	1 (3.10) Thr123 1 (3.24) Phe172	33	35
**11**	Analyticon_NP-014205 Parfumine C_20_H_19_NO_5_	−7.3	−6.9	−111.517	−0.281	−95.502	−0.246	Phe328, Val294, Gln244, Val245, Lys248, Ile295, Pro293	0	24	24	Phe172, Thr123, His124, Phe174, Tyr171, Thr173	1 (3.00) Thr123 1 (3.21) Phe172	21	23
**12**	Analyticon-NP-014522 (2*R*,2′*R,*4a′*S,*6′*S*,7′*R*,8a′*S*)-4,6′,7′-Trihydroxy-2′,5′,5′,8a′-tetramethyl-3′,4′,4a′,5′,6′,7,7′,8,8′,8a′-decahydro- 2′*H*-spiro(furo(2,3-e)isoindole-2,1′-naphthalen)-6(3*H*)-one C_23_H_31_NO_5_	−7.2	−6.9	−97.222	−0.227	−96.182	−0.243	Phe328, Val245, Val294, Gln244, Val245, Lys248, Ile295, Pro293	0	26	26	Phe172, His124, Thr123, Asn136, Arg134, Tyr171, Thr173	1 (2.89A) Thr173 1 (3.08A) Phe172 1 (3.29A) His124 1 (3.11A) Thr123 1 (3.12A) Asn136	21	26
**13**	Analyticon_NP-003228 Isorutarin C_20_H_24_O_10_	−7	−6.4	−105.752	−0.238	−91.824	−0.194	Val245, Asp302, Ile297, Gln241, Val294, Gln244, Val245, Lys248, Ile295, Pro293	1	19	19	Ile82, Phe172, His124, Tyr171, Phe125, Thr123, Thr173,	1 (2.96A) Tyr171 1 (3.13A) Thr123	19	19

Post-docking analysis is representing Binding energies G (kcal/mol), Rescoring binding energies, Per Contact Score (PCS), molecular interactions with EBOV-Z VP35 and VP40 (In bold are common compounds for both viral proteins.)

**Table 4:** Homology modelling of EBOV different strains.

Target Proteins	Ebola Strains	Template PDB ID	Query Cover	E-Value	Maximum Identity
VP40	EBOV-T	3TCQ.A	100%	0	76%
	EBOV-R	3TCQ.A	98%	0	79%
	EBOV-B	3TCQ.A	86%	0	88%
VP35	EBOV-S	3KS4.A	51%	6.00 × 10^−105^	81%
	EBOV-T	3KS4.A	49%	3.00× 10^−102^	82%
	EBOV-B	3KS4.A	49%	2.00× 10^−100^	80%

PSI-BLAST aligned templates against Protein Data Bank (PDB) repository, of EBOV VP35 (*Sudan*, *Tai Forest* and *Bundibugyo*) and VP40 (*Tai Forest*, *Bundibugyo* and *Reston*) with query coverage, E-value, and maximum identity.

## Supplementary Materials

Supplementary materials can be found at www.mdpi.com/1422-0067/17/11/1748/s1.
