# Supplementary material for: Integrated Computational Approach for Virtual Hit Identification against Ebola Viral Proteins VP35 and VP40
Source: Int J Mol Sci. 2016 Oct 26;17(11):1748. doi: 10.3390/ijms17111748 (PMC5133775; doi:10.3390/ijms17111748)
Supplement: Supplementary file 1 [file ijms-17-01748-s001.pdf]

Supplementary Materials: Integrated Computational Approach for Virtual Hit Identification against Ebola Viral Proteins: VP35 and VP40

Muhammad Usman Mirza and Nazia Ikram

Table S1. Autodock vina and DSX score obtained after docking of EBOV-VP35 co-crystalized active compounds against EBOV-Z VP35.

VP35 Inhibitors	PDB ID	AutoDock Vina	DSX-Score
		EBOV-Z VP35 (kcal/mol)	EBOV-Z VP35 (kcal/mol)
VPL57	4IBI	−8	−113.45
VPL58	4IBJ	−7.8	−98.23
VPL48	4IBG	−7.3	−101.321
VPL29	4IBE	−7.2	−98.324
GA017	4IBB	−7.2	−115.2
VPL60	4IBK	−7.2	−97.435
VPL27	4IBD	−7	−102.912
GA246	4IBC	−6.9	−95.82
VPL42	4IBF	−6.8	−6.113

The best autodock vina and DSX score obtained after docking simulations of active compounds against EBOV-Z VP35. The binding energy values are expressed in kcal/mol.

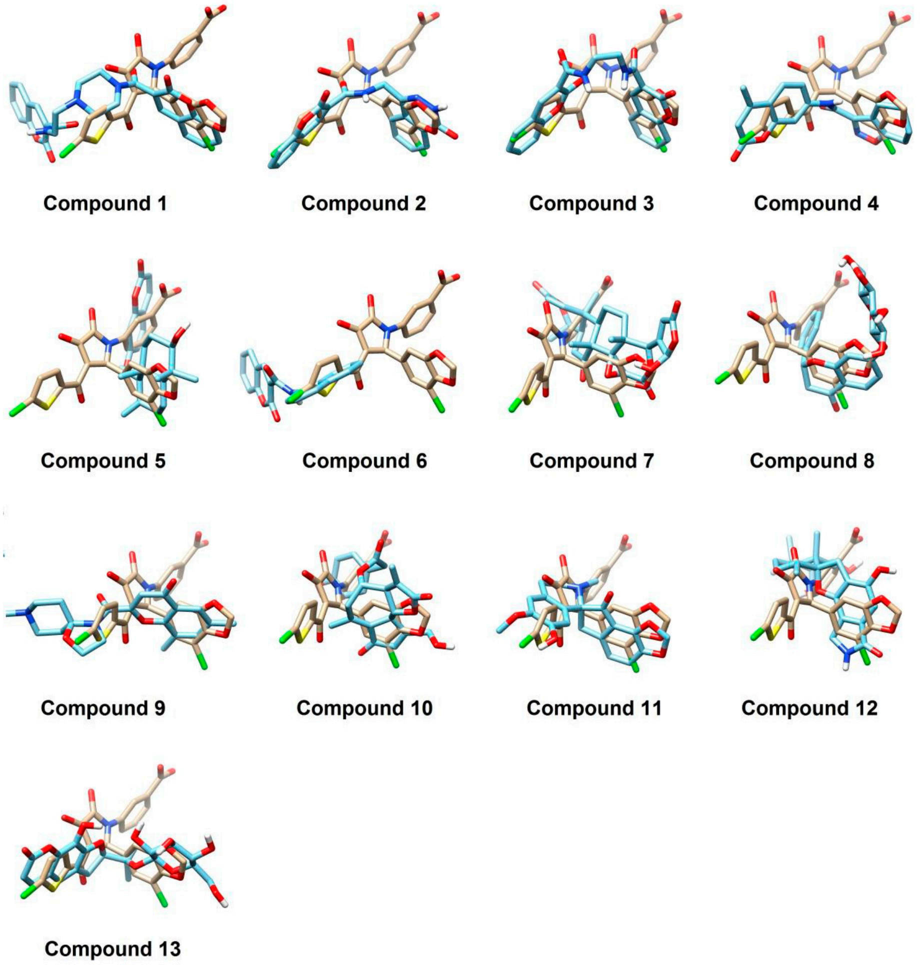

Figure S1. Interactive ligands pose analysis. Superimposition of virtually screen compounds in blue (**1**–**13**) on potent VP35 inhibitor (VPL60) in tan color.
